# Diagnostic approaches for diabetic cardiomyopathy

**DOI:** 10.1186/s12933-017-0506-x

**Published:** 2017-02-23

**Authors:** A. Lorenzo-Almorós, J. Tuñón, M. Orejas, M. Cortés, J. Egido, Ó. Lorenzo

**Affiliations:** 1 Renal, Vascular and Diabetes Laboratory, Instituto de Investigaciones Sanitarias-Fundación Jiménez Díaz, Universidad Autónoma, Av. Reyes Católicos 2, 28040 Madrid, Spain; 2 Department of Cardiology, Fundación Jiménez Díaz, Madrid, Spain; 3 Spanish Biomedical Research Centre in Diabetes and Associated Metabolic Disorders (CIBERDEM) network, Madrid, Spain

**Keywords:** Diabetic cardiomyopathy, Diagnosis, Imaging, Biomarker

## Abstract

Diabetic cardiomyopathy (DCM) is a cardiac dysfunction which affects approximately 12% of diabetic patients, leading to overt heart failure and death. However, there is not an efficient and specific methodology for DCM diagnosis, possibly because molecular mechanisms are not fully elucidated, and it remains asymptomatic for many years. Also, DCM frequently coexists with other comorbidities such as hypertension, obesity, dyslipidemia, and vasculopathies. Thus, human DCM is not specifically identified after heart failure is established. In this sense, echocardiography has been traditionally considered the gold standard imaging test to evaluate the presence of cardiac dysfunction, although other techniques may cover earlier DCM detection by quantification of altered myocardial metabolism and strain. In this sense, Phase-Magnetic Resonance Imaging and 2D/3D-Speckle Tracking Echocardiography may potentially diagnose and stratify diabetic patients. Additionally, this information could be completed with a quantification of specific plasma biomarkers related to related to initial stages of the disease. Cardiotrophin-1, activin A, insulin-like growth factor binding protein-7 (IGFBP-7) and Heart fatty-acid binding protein have demonstrated a stable positive correlation with cardiac hypertrophy, contractibility and steatosis responses. Thus, we suggest a combination of minimally-invasive diagnosis tools for human DCM recognition based on imaging techniques and measurements of related plasma biomarkers.

## Diabetes and cardiovascular risk

Cardiovascular pathologies are the most common cause of mortality in the world, and diabetes mellitus is one of the major risk factor for cardiovascular disease development [1]. By 2025, diabetes is expected to affect 300 million people with a prevalence of 5.4%. According to the Framingham Heart Study, the risk of heart failure in diabetes is increased 2.4-fold in men and fivefold in women compared to non-diabetic population [2]. In this regard, though cardiovascular diseases debut approximately ten years later in women than in men, in diabetes, that ‘female protection’ may not defend from cardiovascular injuries, suggesting a key role of sexual hormones in diabetic cardiomyopathy (DCM) development [3].

The relationship between diabetes and heart disease has been known for many years, but the first description was made by Leyden et al. in 1881 [4], when he stated that heart failure (HF) was a *“frequent and noteworthy complication of diabetes mellitus”.* Later in 1972, Rubler et al. introduced the entity named DCM, in order to distinguish this condition from other types of cardiomyopathy [5]. Nowadays, the minimal criteria to be diagnosed with DCM include left ventricular diastolic dysfunction and/or reduced left ventricular ejection fraction (EF), pathological left ventricle hypertrophy, and interstitial fibrosis [6]. This left ventricular remodeling may be presented as dilated eccentric with left ventricular systolic dysfunction or concentric with left ventricular diastolic dysfunction, being the later the most frecuent observed by general cardiologist and diabetologist [7]. DCM is defined as a ventricular dysfunction observed in diabetic patients, independently of coronary artery disease, valve disease or hypertension. Also, DCM may be present in up to 60% normotensive patients with diabetes as a pseudonormalization of diastolic pattern, which is an advanced left ventricular diastolic dysfunction characterized by an intermediate stage between impaired relaxation and restrictive filling with reduced myocardial contractility and strain [8]. Finally, DCM can be also present as a diminished contractile function while exercising [9].

DCM affects approximately 12% diabetic patients, disturbing almost 22% subjects over 64 years-old, which represent between 36 and 66 millions of diabetic people, respectively [10]. However, vascular pathologies frequently coexist with DCM. The accelerated atherosclerosis in T2DM may be explained by several factors including low-grade inflammation, metabolic disorders such as hyperglycemia with increased formation of advanced glycation end-products (AGEs), obesity, dyslipidemia, hyperinsulinemia, oxidative stress, and autonomic imbalance. Macroangiopathy complications include heart disease, stroke and peripheral arterial disease [11], whereas microangiopathy comprise retinopathy [12], neuropathy [13], and nephropathy [14]. In this sense, a clear association between myocardial strain and albuminuria has been observed in T1DM patients [15], and protective effects of SGLT-2 inhibitors on kidney have been also seen for HF prevention in T2DM individuals [16]. In addition, DCM concurs with cardiovascular risk comorbidities such as hypertension and overall obesity, which complicates its diagnosis and specific treatment [17]. A major risk factor for the development of T2DM is obesity, and according to the World Health Organization, over 600 million adults worldwide were obese as of 2014 [18]. Since lipids can accumulate in the hearts of both obese and T2DM subjects, subsequent metabolic disbalance and lipotoxicity are major mechanism contributing to diabetes-related ventricular dysfunction. Also, obesity-induced hyperinsulinemia stimulates hepatic expression of lipogenic genes, de novo lipogenesis and the secretion of VLDL lipoproteins [19]. In type-II diabetes (T2DM), the coexistence with these cardiovascular risk factors and insulin resistance highlights the different pathogeny with type-I diabetes (T1DM), which is more related to genetic factors.

## Cardiac responses to diabetes

**Fig. 1:**
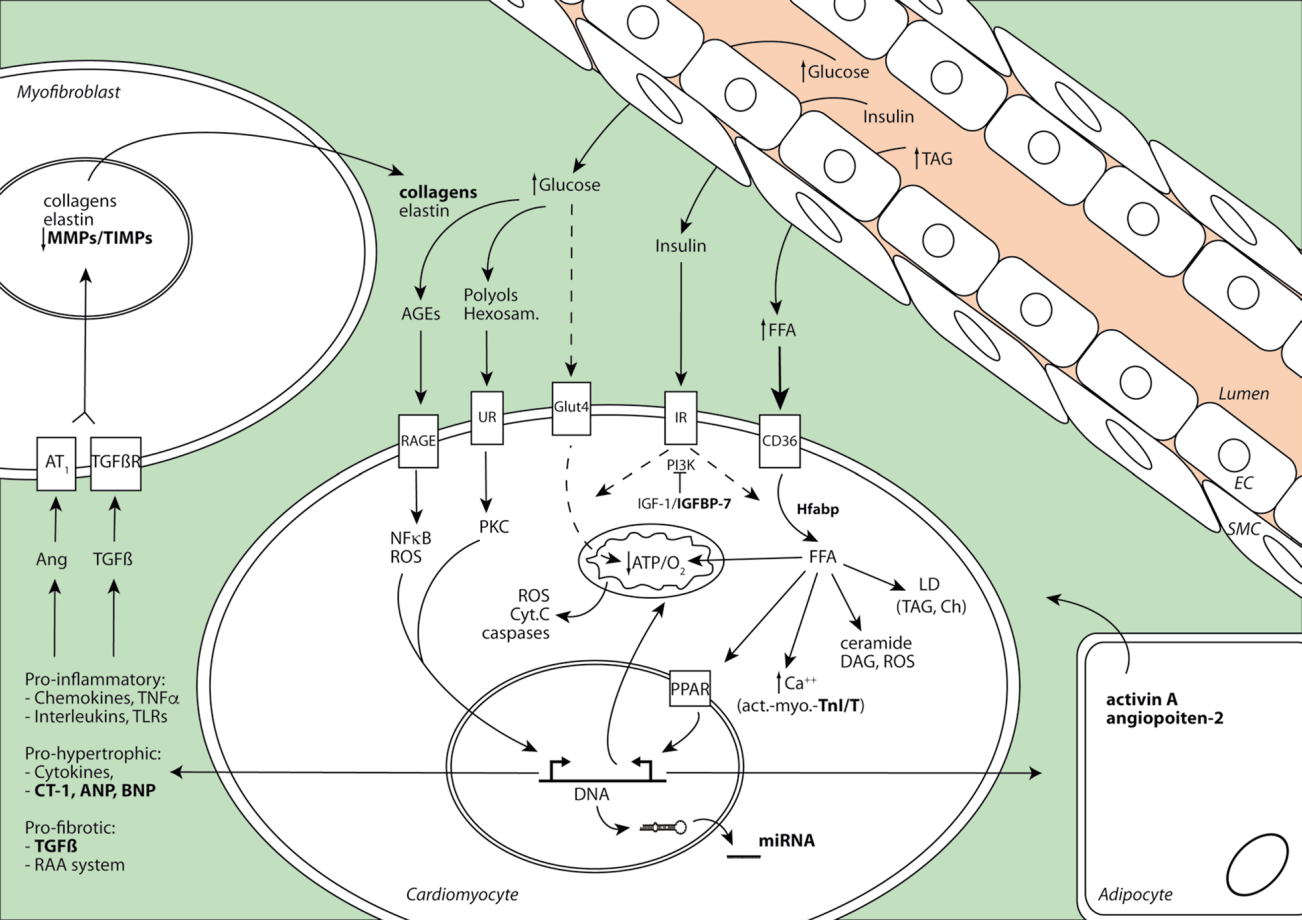
Cardiac scenario in diabetic patients. Biomarkers discovery for DCM diagnosis. In diabetes, high levels of blood glucose and fatty-acid, together with a defect in insulin signalling (insulin resistance) activate diferent cellular mechanism in the myocardium. Glucose cannot be appropriately assimilated by cardiomyocyte and deviate to glucose metabolites such as AGEs (with ECM proteins), polyols and hexosamine, which may activate pro-oxidant and pro-inflammatory responses. Energy relies on FFA only, which are uptaken in excess and accumulated as secoundary toxic products such as TAG (in lipid droplets; LD), ceramide and DAG, leading to steatosis. They also decrease calcium flux between sarcoplasmic reticulum and cytosol, reducing actin-myosin-TnI/T complexes, and contractibility. FFA also bind PPAR receptors for upregulation of mitochondrial beta-oxidation enzymes, which produce non-efficient ATP and ROS, triggering mitochondrial dysfunction (release of cytochrome-C) and apoptosis. All these stimuli promote the expression specific miRNAs, and pro-hypertrophic and pro-fibrotic factors such as RAA and TGFβ systems, which may play autocrine and paracrine roles on (myo)fibroblast and adipocytes (from EAT). Interestingly, some of these molecules (in *bold*) can be released to the circulation and being used as biomarkers of cardiac dysfunction. *UR* unspecific receptor, *TLRs* toll-like receptors

There are different structural and functional modifications in the myocardial tissue after diabetes. These events emerge from metabolic alterations induced by hyperglycemia, insulin resistance and hyperlipidemia (Fig. 1). The lack of insulin sensitivity and glucose assimilation in the heart is evident by the reduction in number of glucose transporters Glut1, and mainly, Glut4. In this line, impaired fasting glucose and left ventricular diastolic dysfunction have been correlated in middle-age adults [20]. As a compensatory mechanism, fatty acid transporters are increased, and most ATP generation relies on fatty acid degradation [21]. However, fatty acid may saturate ß-oxidation and accumulate in the cytosol, leading to lipotoxic effects by generation of ceramides, diacylglycerol and reactive species of oxygen (ROS). In this sense, hyperglycemia elicits also ROS and advanced glycation product (AGE) formation, which lead to cardiac glucotoxicity. Both, the lack of fuel and lipo/gluco-toxicity are promoters of calcium misbalance, mitochondrial/endoplasmic reticulum failure and apoptosis, triggering cardiac low-grade chronic inflammation, fibrosis and contractile dysfunction [22]. Among others, the renin-angiotensin-aldosterone (RAA) and TGFß systems, together with specific cytokine/chemokine production, are remarkably enhanced.

Several animal models have somehow mirrored human DCM to further deep into the molecular mechanisms and key mediators [23]. Diabetic rodents have been widely studied because of their natural resistance to atherosclerosis [24]. T1DM can be harshly induced by toxins (i.e., streptozotocin, alloxan) and genetic modifications (i.e., calmodulin, insulin), whereas T2DM is mostly reproduced by diets (i.e., high-fatty) and genetic (i.e., leptin system) mutations. All of them showed cardiac abnormalities, including myocyte hypertrophy and myocardial inflammation and fibrosis. An increase in steatosis and apoptosis have been also seen, reflecting a multifactorial pathogeny in the disease [25]. In humans, T1DM-associated cardiac dysfunction has been mostly related to hyperglycemia, oxidative stress and subsequent myocardial fibrosis. Instead, cardiac dysfunction in T2DM has been linked to hyperinsulinemia, insulin resistance and coexistence of obesity, dislipemia and/or hypertension, promoting cardiomyocyte hypertrophy and steatosis. Thus, since T1DM may exhibit better glucose control and absence of comorbidities, cardiac dysfunction can be more severe in T2DM [26]. In this sense, T1DM patients improved cardiac indexes after intensive glucose control [27]. However, intensive glycemic goals (HbA1c < 7%) have failed to prevent cardiac complications in long-term diabetic patients or have even increased cardiovascular mortality [28, 29]. The failure of the therapy approaches employed to date calls for accelerating efforts towards the development of new therapeutic strategies capable of preserving heart function while contributing to the overall care of diabetes. In this regard, an early and specific detection of DCM could be critical for therapeutic success avoiding further HF and fatal fate.

## Diagnosis of diabetic cardiomyopathy

Diastolic dysfunction is defined as a defect in ventricular relaxation in that leads to increase pressures and a subsequent impaired filling during diastole. Systolic dysfunction is the inability of the myocardium to eject the adequate blood volume, what it means, heart exhibits a low EF. Diastolic dysfunction is twice more common in diabetic than non-diabetic subjects, and appears earlier [30]. Also, age can influence detection of diastolic dysfunction in DM patients, and thus, this deficiency should be judged for every age. At initial stages of DCM, left ventricular EF could be preserved, whereas diastolic dysfunction with reduced diastolic filling, prolonged isovolumetric relaxation and increased atrial filling, is established. Relaxation abnormalities at the heart are observed concurrently with autonomic neuropathy, and are frequently related to hyperglycaemia [31]. In addition, a decrease in longitudinal systolic function and a compensatory increase in radial function can be also present. More than 25% of diabetic patients showed systolic strain abnormalities preceding diastolic dysfunction, which may be influenced by risk factors such as hypertension or obesity, for clinical interpretation and imaging quality [14, 30]. However, DCM is currently detected at late stages of the pathology through identification of systolic dysfunction, when HF has been already instituted [32]. Diabetic patients arriving to emergency departments with symptoms of HF are examined by non-invasive tests such as chest X-ray, to assess fluid accumulation in the lungs, electrocardiography, to identify ventricular overload, and conventional cardiac ultrasound, to assess structural and functional abnormalities of the myocardium. Additionally, natriuretic peptides plasma levels may also be of help in the diagnosis of heart failure. Other biomarkers not used in the clinical practice in this setting, could provide also additional information. These biomarkers can be released after a wide variety of cardiac and/or skeletal muscle injuries such as inflammation (i.e., C-reactive protein), hypertrophy/stiffness and necrosis (i.e., troponins) in relation to different diseases such as myocardial infarction, arrhythmia, myocarditis, hypertension, or any secondary cardiac injury (i.e., chemotherapy, renal kidney disease). Thus, recent human and mainly pre-clinical data suggest new techniques for detection of premature DCM based on imaging and biomarkers analysis.

### Approaching to DCM diagnosis by imaging technologies

An accurate method to diagnose DCM could include endomyocardial biopsy sampling [33]. The morphological features observed in the biopsy, usually taken from interventricular septum to avoid complications of transmural procedure within the free wall (i.e., perforation or arrhythmias), may be considered as representative of the whole cardiac tissue. Kumar Das et al. biopsied the heart of a group of hyperglycemic and asymptomatic or symptomatic T2DM patients [34], correlating the metabolic alterations with the different ultrastructural changes. Indeed, collagen [35] and fat [36] deposits were found in left ventricular transmural biopsies from T2DM patients with no evidence of hypertension or coronary artery disease. Also, endomyocardial biopsies from symptomatic T2DM patients with HF exhibited higher loss of myofibrils than in asymptomatic T2DM patients by electron microscopy [37]. However, due to its invasive nature and high potential risks, this approach has been relegated to research studies. Similarly, a classic method to characterize cardiac dysfunction in DCM may include cardiac catheterization. In this method, a catheter is introduced through the radial or femoral artery up to the left ventricle to directly measure cardiac pressures and motility. A coronary angiogram may be performed in parallel to exclude coronary artery disease. An end-diastolic pressure over 16 mmHg in the left ventricle may confirm diastolic dysfunction [38]. However, this methodology is invasive and not specific to diagnose the DCM-associated diastolic dysfunction. Then, non-invasive techniques are preferred for this purpose.

#### Echocardiography

**Fig. 2:**
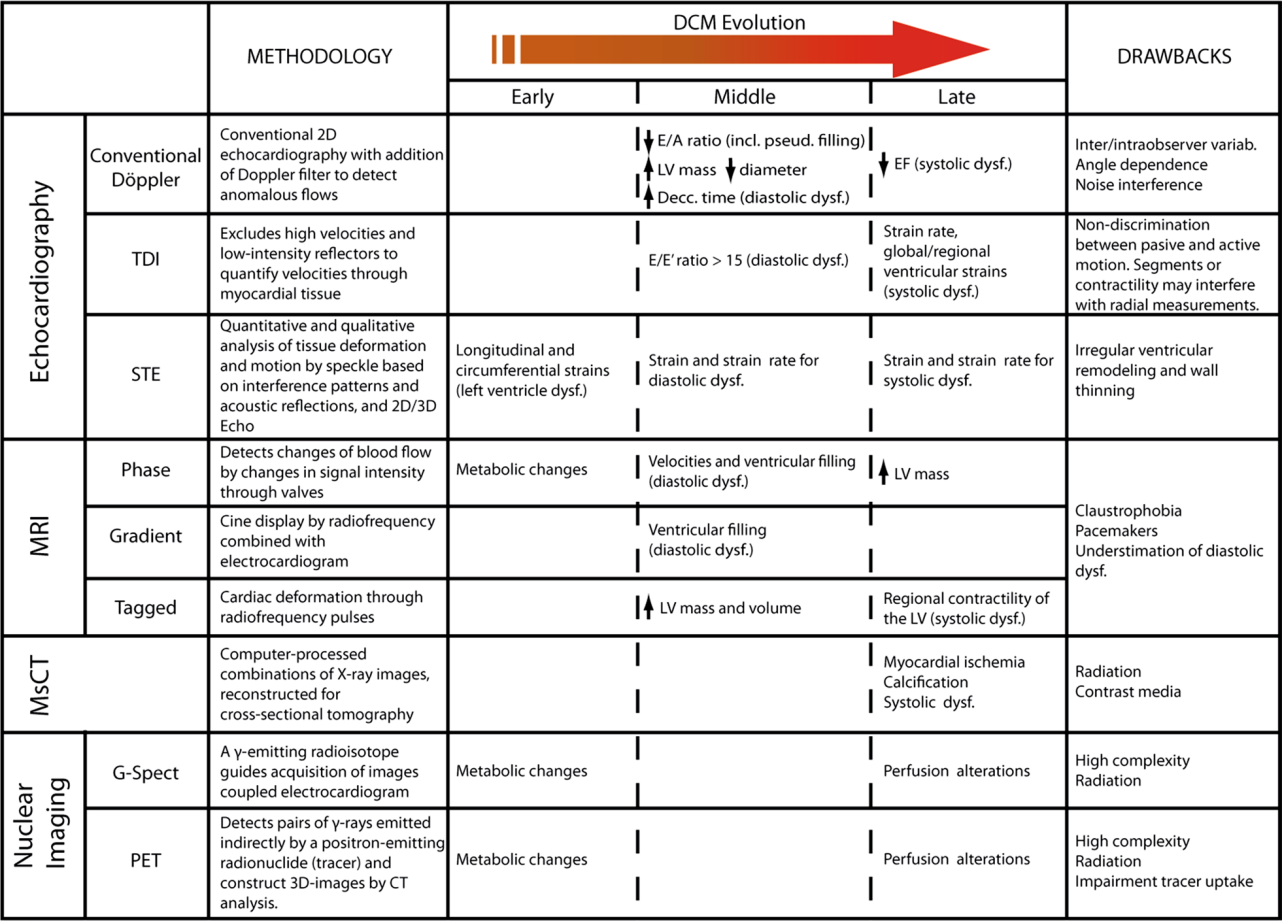
Main imaging approaches for DCM identification. Several methodologies can be addressed to both T1DM and T2DM patients for evaluation of cardiac dysfunction. Early, middle or late responses of DCM may be detected. However, some inconvenient must be contemplated to personalize suitable strategies

Nowadays, this non-invasive approach is the gold standard diagnostic tool to identify structural cardiac disorders. It provides a reliable identification of the structural abnormalities seen in early stages of DCM, such as impaired diastolic filling and left ventricle hypertrophy. It can also be helpful for the assessment of the progression of the disease and evaluation of treatments [39]. Echocardiography has been widely studied in rodent models even without using anesthesia, and thus, eluding the cardiodepressant effect [40]. Several modalities can be used (Fig. 2).

##### Conventional Döppler-echocardiography

Two-dimensional (2D) echocardiography allows to examine cardiac structures and their movement. Furthermore, Döppler technology, that is based on the shift in frequency of ultrasound signals reflected from moving objects, is useful to study blood flow [41]. In addition to detecting anomalous flows, such as those seen in valve regurgitation, or to estimate the severity of valve stenoses, it may be of help to analyze diastolic function. Some manoeuvres such as Valsalva could be also used with trans-mitral Döppler signals to increase accuracy [42]. In this regard, ventricular diastolic filling velocities are known to be altered in the presence of diastolic dysfunction. A reduction of the E-wave, which represents the early ventricular filling, or a raise in A-wave, that corresponds to the atrial-driven ventricular filling (decreased E/A ratio), as well as a prolonged deceleration time of the E wave are signs of relaxation impairment, and have been observed in T1DM patients [27]. Similarly, a high incidence of diastolic dysfunction has been demonstrated in asymptomatic T2DM patients by this approach [43]. Moreover, other early DCM-associated abnormalities in T1DM and T2DM subjects, such as left ventricular hypertrophy, could be also detected with 2D-Echo-Döppler [44]. Importantly, in diabetic primates 2D-Echo-Döppler detected both diastolic (by decreased E/A ratio and prolonged deceleration time) and systolic (by decreased EF) dysfunction, accompanied by left atrial hypertrophic remodeling [45]. In T1DM rodents, Wichi et al. found similar results using 2D-Echo-Döppler or catheterization in relation to diastolic and systolic left ventricle function [46]. Also, in T2DM mice, 2D-Echo-Döppler successfully detected early changes in diastolic function and myocardial hypertrophy [47, 48]. Additionally, 2D-Echo-Döppler can be used to detect epicardial adipose tissue (EAT) accumulation in DCM. EAT can correlate with visceral adipose tissue and thus, is increased in obesity. EAT is an important player in the physiological and biochemical regulation of cardiac homeostasis by release of proinflammatory cytokines such as interleukins, monocyte chemoattractive protein-1, leptin, plasminogen activator inhibitor-1, resistin, and TNFα. Although EAT thickening could be related to other cardiomyopathies such as ischemic cardiac disease, an enlargement of EAT has been observed by 2D-Echo-Döppler in both T1DM [49] and T2DM [50] patients in correlation with HF and cardiac biomarkers (i.e., BNP, troponin-T and C-reactive protein). However, some limitations for 2D-Echo-Döppler, such as inter/intra-observer variability, angle dependence or noise interference, have to be considered. This approach may be also influenced by heart rate and volume status. Also, the pseudonormal filling pattern in diabetic patients could be underestimated [51].

##### Tissue Döppler imaging (TDI)

Since tissue has a greater reflectivity and slower motion that blood flow, TDI applies filters that exclude high velocities and low-intensity reflectors (that is, the red blood cell motion) to quantify velocities through myocardial tissue. Thus, TDI allows measurements of motion during cardiac cycle,analogous to the conventional Döppler [51]. In this regard, it is important the diastolic mitral annular motion analysis: E′ wave (early diastolic velocity) and A′ wave (atrial-driven motion) The E/E′ ratio (early transmitral velocity to TDI mitral annular early diastolic velocity) has been described as an indirect measure of diastolic dysfunction when it is over 15 [7]. Furthermore, the strain rate, and global and regional ventricular strains can be analysed with TDI. Both parameters provide a more direct assessment of intrinsic myocardial contractility. With strain and strain rate, myocardial velocities in different myocardial segments are evaluated, independently of pre-load changes and providing an extra advantage over 2D-Echo-Döppler. Longitudinal, circumferential and radial cardiac contractions are examined in myocardial fibres, allowing the quantification of ischemia and fibrosis. In this regard, in forty recently-diagnosed T2DM patients, 50% of them were diagnosed with TDI as early-staged DCM [41]. TDI with Valsalva was also useful for detection of diastolic dysfunction with pseudonormal filling patterns in T2DM monkeys [52]. Although heart rate and circumferential/torsion cardiac movements in rodents are different than in humans, an impairment in radial direction rather than in longitudinal was detected in T1DM rats as a marker of fibrosis [53]. However, TDI achieves absolute tissue velocity but cannot discriminate passive motion (related to translation or tethering) from active motion (fiber shortening or lengthening). Also, active segments or compensatory contractility may interfere with radial measurements. Moreover, quantification is limited to the segments in which deformation aligns with the ultrasound beam [54].

##### Intravenous contrast echocardiography (ICE)

This procedure is based in the increased reflectivity of intravenous contrast agents (microbubbles) due to the differential reflection of the contained gas compared with surrounding blood and tissue.Thus, ventricular opacification enhances endocardial borders by delineating anatomy, leading to higher accuracy of ventricular size and, more importantly, ventricular motion [55]. The addition of ICE to 2D-echocardiography showed an improvement in the accuracy of left ventricular function evaluation in a non-selected cardiac population [56]. Although ICE has not been tested in human diabetes, the alteration of contractility and systolic function, as well as microcirculation, were demonstrated in T1DM rats [57]. However, most studies performed in DCM assessed myocardial blood flow instead of motion, and the intravenous administration of contrast media may also limit its use. Moreover, not all endocardial segments can be correctly visualized [55].

##### Döppler acoustic echocardiography

This modality of Echo is focused on the acoustic properties of the heart, which are modified when fibrosis is present. A decrease in cyclic variation index allows to diagnose early fibrosis, and also, hypertrophy. Although there are no data in experimental DCM, Pérez et al. [58] characterized the myocardial acoustic properties in T1DM patients. They found that cyclic variation of integrated backscatter was reduced and delayed among diabetic subjects. Indeed, patients with diabetic complications such as neuropathy or retinopathy had the greatest reduction in cyclic variation and the highest increase in delay [58]. These data were confirmed in other clinical study in T1DM patients as an intensification of myocardial echo-density defined by the integrated backscatter index [59]. However, the mechanisms of cyclic variation of integrated backscatter are not completely understood and may be a reflection of myocardial physiology rather than biochemical changes assessed by acoustic ultrasound [43].

##### Three dimensional (3D)-echocardiography

Current generation 3D-Echo allows for collection of a full pyramidal data set in real time (opposite to the triangular data set of the 2D technique). 3D-Echo provides several advantages over conventional ultrasound methodology since it can evaluate ventricles with abnormal shapes or mobility. It also overcomes disadvantages of 2D-Echo by reducing calculation errors and interferences, and increasing the visualization [60]. 3D-Echo improves anatomical localization and measurement accuracy with the use of tomographic images and strain techniques, giving information of both diastolic and systolic dysfunction [61]. Although there are no data in experimental DCM, subjects with prediabetes or T2DM exhibited by 3D-Echo a right ventricular and atrial deformation, and cardiac dysfunction [62]. However, high quality imaging and appropriate heart rate is a prerequisite to accurately measure cardiac function. Thus, the assessment of regional wall segments could be challenging under stress conditions [63].

##### Speckle tracking echocardiography (STE)

STE is a relatively new technique to achieve myocardial mechanical deformation (strain and strain rate). STE overcomes major drawbacks of conventional techniques (i.e., TDI) such as inter/intra-observer variability, angle dependence or noise interference [64]. It uses sequence of images obtained from 2D- or 3D-echocardiography and quantifies the distance between pixels during the cardiac cycle. Thus, alterations in myocardial deformation in the three axes (radial, circumferential, and longitudinal strain) are detected. In this sense, strain and the strain rate are solid indexes for left ventricular contractility, and more independent of pre/after-load than EF [65]. 3D-STE successfully confirmed the correlation between diabetic microangiopathy and myocardial deformation in asymptomatic T2DM patients by reduction of global longitudinal and circumferential strains [66]. In this regard, 24% diabetic patients were diagnosed with impaired systolic function by 2D-STE, but not by other conventional strategy [67]. Importantly, a decreased longitudinal systolic strain detected by STE was associated with cardiovascular events and provided incremental prognostic value up to ten years after revealing [68–70]. Also, longitudinal strain and subsequent increased torsional deformation have been crucial to diagnose early myocardial disease in T1DM patients [71]. In asymptomatic T1DM, 2D-STE also demonstrated subclinical left ventricular dysfunction and right systolic dysfunction [72]. In pediatric T1DM, 2D-STE showed impaired longitudinal and circumferential strain as signs of hyperdynamic left ventricular contractility early in the course of the disease [73]. In addition, 2D/3D-STE have been found to be highly reliable in animals [74]. In sheep, 2D-STE provided earlier information about left ventricular function. Specifically, left ventricle free wall was affected in both short and long-axis, whereas the strain and the strain rate were altered in the radial axis [75]. In early stage T2DM rats, 2D-STE detected left ventricular deformation associated with cardiomyocyte Ca^2+^ transients delay [76]. In T1DM rabbits, longitudinal and circumferencial strain gradually diminished from endocardium to epicardium, which was consistent with invasive labelling studies [77]. Recently, in T1DM mice, 2D-STE showed early alterations such as systolic radial strain, radial strain rate, radial displacement, and radial velocity, as well as decreased circumferential and longitudinal strain rate during the progression of disease and earlier than contractile changes detected by 2D-Echo [78]. However, as a weakness, irregular ventricular remodeling and wall thinning may affect STE accuracies.

#### Magnetic resonance imaging (MRI)

Cardiac MRI can be also useful for DCM diagnosis. In contrast to echocardiography, MRI operates with greater spatial and temporal resolution to evaluate chamber size, left ventricular EF and myocardial mass distribution. MRI provides extra information about myocardial fibrosis and subclinical ischemia, as premature parameters of cardiac dysfunction. There are two main pulse-sequence MRI defined by the relaxation properties of the different tissues, known as T1 and T2 weighted, that allow the characterization of the tissues. In T1-weighted imaging, tissue regions with high fat content or fibrosis appear bright, whereas in T2-weighted imaging those areas with an increase in the water content look bright. Thus, MRI attains also higher characterization of regional and local systolic contractility [27], through several modalities.

##### Gradient-Echo-MRI

Gradient-Echo-MRI creates a cine display about different moments of the cardiac cycle by combining radiofrequency pulses with electrocardiogram [32]. Thus, MRI provides also higher characterization of regional contractility of the left ventricle [27], and has been used to determine right ventricle dimensions and function, which were found to be impaired in men with uncomplicated T2DM [79].

##### Phase-contrast-MRI

Phase contrast imaging is an MRI technique that can be used to visualise moving fluid and quantify the velocity in a certain area (myocardial valves). Mitral valve inflow velocities, early deceleration time, and pulmonary vein flow velocities are diastolic parameters that can be measured by phase-contrast MRI. Phase-contrast-MRI can obtain images under any circumstance without influence of breathing and minimal variability [80]. The direction and velocity of flow, as well as turbulences, provide information about intra-myocardial velocities and hypertrophy [32]. Although there are not data in preclinical diabetes, this procedure revealed diastolic dysfunction in association with low levels of myocardial phosphocreatine to adenosine-triphosphate (PCr/ATP)-ratio, and impaired cardiac metabolism in T1DM patients [81]. Also, phase-contrast MRI revealed left ventricular dysfunction in well controlled T2DM normotensive patients [82].

##### Tagged-MRI

This technology uses radiofrequency pulses of the myocardium to determine cardiac deformation along cardiac cycles through strain rates and torsion recoveries [83]. Tagged-MRI indicated higher left ventricular torsion with normal EF and left ventricular mass in well controlled T1DM patients [84]. Similarly, by tagged-MRI coupled to 3D-Echo, a decrease in circumferential and longitudinal strains and strain rates was found among T2DM patients with poor glycemic control and presence of other cardiovascular risk factors [85]. These data were not confirmed in well-controlled T1DM patients, suggesting a detrimental role of comorbidities and metabolic disturbances in cardiac strain [84]. In experimental T1DM, tagged-MRI showed an increase of left ventricular volumes and mass, and left ventricular hypertrophy and fibrosis, in parallel to metabolic alterations [86, 87]. In addition, T1 mapping incorporated on MRI can provide an alternative method of quantification of diffuse non-specific fibrosis [88]. In asymptomatic T2DM subjects, an early correlation between myocardial dysfunction (detected by Echo-Döppler) and an enhanced T1 mapping was disclosed [88].

However, MRI may not be available to all patients, and has some limitations. It may underestimate diastolic dysfunction, cannot be compatible with some pacemakers or implantable desfibrilators, and may produce claustrophobia in some patiens. Also, in animals, MRI requires anesthesia, which could interfere with cardiac performance [24].

#### Multi-slice computed tomography (MsCT)

MsCT uses volumetric methods to obtain ventricular function parameters. End-systolic and -diastolic volume are calculated pixel-by-pixel in different cardiac rotations arranged and reconstructed by an automated software. Small segments of data are collected along several cardiac cycles to generate a final image of computed tomography [89]. High temporal resolution is needed to obtain artefact-free images during systole and diastole. Moreover MsCT has been proved as a promising tool for ischemic cardiac disease by estimation of coronary artery calcification and atherosclerosis. Although there are no data in experimental DCM, in T2DM patients, MsCT could be a reliable assessment of left ventricular dysfunction [90]. However, radiation exposure and the use of contrast media along with side effects may relegate diagnosis of DCM to other techniques [91].

#### Nuclear imaging

These techniques focus on cardiac autonomic dysfunction, as a common finding in early stages of diabetes, as occurs during impaired glucose tolerance, where heart rate and myocardial blood flow may be disturbed.

##### Gated-SPECT (G-SPECT)

G-SPECT provides simultaneous assessment of cardiac perfusion and left ventricular function by using labeled myocardial perfusion agents, providing real information about ventricular function in 3D, wall thickening, motion, and diastolic parameters [92]. G-SPECT, with either Tl-201 or Tc-99m tracers, was considered a reliable assessment of left ventricular global and regional function in non-diabetic patients compared to 2D-echocardiography. Furthermore, this technique seemed to be highly reproducible, when compared to conventional echo [93]. However, there is not data in human or experimental DCM, but G-SPECT might be especially useful in these patients, as the metabolism of tracers depends on metabolic alterations found in the disease [94].

##### Positron emission tomography (PET)

Similarly, PET scan has been proposed to diagnose DCM, in special, for those subjects with obesity or advanced left ventricular dysfunction and coronary artery disease, who cannot be analysed by G-SPECT [95]. Asymptomatic T2DM patients exhibited an increase in myocardial fatty acid metabolism by PET, though no relation was found between diastolic dysfunction and energy metabolism parameters [96]. In a rat model of early DCM, an small-animal PET using ^18^Fluoro-desoxyglucose as a tracer, revealed a decrease of myocardial glucose utilization and increased fatty acids oxidation, in association with impaired myocardial function [97]. In T2DM rats, PET showed alterations in myocardial glucose utilization that were linked with cardiac dysfunction [98]. However, ^18^Fluoro-desoxyglucose itself may constitute a drawback for diabetic patients with impairment in glucose uptake.

### Approaching to DCM diagnosis by biomarkers detection and quantification

Several efforts have been made to improve DCM detection by non-invasive quantification of biomarkers in body fluids such as urine, saliva, semen or tears, but all of them were not conclusive [99]. However, by minimally invasive blood sampling, plasma biomarkers have headed consistent and stable results. Cardiac autocrine, paracrine or excreted molecules could be released from the diabetic heart in response to damage, following (mal)-adaptive mechanisms associated to structural and functional alterations. Chronic, late or irreversible events such as inflammation or apoptosis, detected by markers as C-reactive protein or Creatine kinase-MB, may not provide valuable information. However, detection of early and reversible responses such as hypertrophy, contractibility, steatosis or even fibrosis by related factors might be optimal for DCM diagnosis.

#### Myocardial pro-hypertrophic biomarkers for DCM

Non-physiological cardiac hypertrophy is adaptive and compensatory in DCM. This cellular growth presents as increased myocardial cell surface area, protein synthesis and reactivation of foetal genes (such as atrial natriuretic peptides and β-myosin heavy chain). However, in the long term, hypertrophy leads loss of function and death of cardiomyocytes, favoring HF. Thus, a premature detection of pro-hypertrophic factors could be helpful to diagnose early DCM (Fig. 1). In this sense, the family of natriuretic peptides that comprises the atrial natriuretic peptide (ANP), brain natriuretic peptide (BNP), and its biologically inactive fragment N-terminal proBNP (NT-proBNP), has been used as a HF biomarker. The half‐life of BNP is higher than that of ANP, raising 20 min, whereas NT‐proBNP is degraded after 120 min. These neurohormones are secreted by the atrial and ventricular myocardium in response to HF [100]. Interestingly, association between HF and DM was blunted due to the considerable effect of BNP in counteracting insulin resistance [101]. ANP is stored in granules and can be released immediately after stimulation, but only small amounts of BNP are stored, being its rapid gene expression the underlying mechanism that stimulates its secretion [102]. Biological actions of these cardiac hypertrophic markers are mediated through specific membrane-bound receptors coupled to cGMP signaling. Both ANP and BNP promote diuresis, natriuresis, hypotension and smooth muscle relaxant activities.

In T1DM patients, high circulating NT-proBNP levels identify left ventricular systolic dysfunction in 96% cases [103], whereas ANP was elevated after early diastolic dysfunction and cardiac sympathetic dysinnervation [104]. However, both peptides were useful sensors for diastolic dysfunction in diabetic patients who were symptomatic, had a restrictive filling pattern or pseudo-normalized mitral flow pattern, but not in those subjects asymptomatic or with relaxation abnormalities [100]. Another studies also demonstrated elevated levels of NT-proBNP in diabetic patients with diastolic dysfunction and uncontrolled diabetes (HbA1c > 7%), but not in asymptomatic diabetic patients with mild diastolic dysfunction [105, 106]. Moreover, NT-proBNP played a prognostic rather than a diagnostic role in another population of diabetic patients [107]. In experimental DCM, natriuretic peptides have been able to predict HF. Both NT-proBNP and ANP were significantly increased in plasma and atrial muscle of T1DM rats [108, 109]. Studies in pre-diabetic rats demonstrated that NT-proBNP levels could diagnose left ventricle hypertrophy [110]. Plasma NT-proBNP was significantly higher in T2DM with HF rats [111]. Nevertheless, natriuretic proteins are also secreted in response to acute myocardial infarction, unstable angina and increased wall pressure/volume overload [100].

Thus, new prospective pro-hypertrophic biomarkers are needed for DCM detection. In this regard, cardiotrophin-1 (CT-1), a member of the gp130 cytokine family, is mainly released from cardiomyocytes after oxidative and mechanical stress or RAA system stimulation [112] (Fig. 1). CT-1 can modulate cardiac hypertrophy, contractility, fibrosis and ischemia through reduction of cell proliferation, apoptosis, oxidative stress and inflammation, by activation of JAK/STAT and MAPK pathways [112, 113]. However, chronic activation of CT-1 promotes cardiovascular remodelling (i.e., cardiac hypertrophy) and HF In cardiomyocytes of neonatal rats [114, 115]. Interestingly, CT-1 is also a key regulator of cardiac glucose metabolism by increasing insulin-stimulated glucose uptake [116]. In this sense, higher plasma CT-1 levels positively correlated with basal glycaemia and left ventricular hypertrophy in T2DM patients [117]. Also, patients with impaired glucose tolerance or recently diagnosed diabetes exhibited elevated CT-1 plasma levels [118], but intriguingly, low concentrations were found in non-diabetic obeses and overweighted [97]. However, expression of CT-1 is not exclusive for the heart, and high levels of CT-1 have been also found in different cardiomyopathies including ischemia.

#### Myocardial contractile biomarkers for DCM

Troponins have been related with myocardial contractility in different cardiomyopathies. This multiprotein complex is constituted by Troponin I (TnI), C (TnC) and T (TnT) in all striated muscles. Troponin controls calcium mediated interaction between actin and myosin (Fig. 1). In particular, TnC is the calcium-binding subunit of the complex and its interaction with TnI and TnT is crucial for cardiac contraction [119]. The myocardial-specific isoforms TnI and TnT are sensitive markers of necrosis, and thus, are widely used in the routine clinical practice [120]. Interestingly, TnI and TnT were found to be phosphorylated in human myocardial biopsies, which may lead to depressed myofilament function and calcium-sensitivity [121]. Data have been reported in non-adult population. Newborns of diabetic women showed a significant correlation between plasma TnI levels and decreased left ventricular end diastolic diameter and increased interventricular septum [122]. Circulating TnT was also high in infants born from T1DM mothers [120]. In experimental models of DCM, particularly in T2DM rats with HF, plasma Tn-T were notably higher [111].

Thus, new prospective biomarkers related with the contractile function of the cardiomyocyte are needed for DCM detection. For this purpose, EAT can release autocrine and paracrine molecules (Fig. 1). Interestingly, isolation of these secreted factors from TDM2 patients induced contractile dysfunction and insulin resistance in primary rat cardiomyocytes [123]. In particular, activin A and angiopoietin-2 stimulated insulin-mediated phosphorylation of Akt, a key regulator of myocardial glucose uptake [124]. Activin A, belonging to TGFβ family, was released from cultured EAT biopsies from T2DM patients inhibited insulin action via Akt pathway blockade [125]. Similarly, in high-fat feeding guinea pigs, where EAT-secreted molecules increased activin A-signalling, decreased calcium ATPase-2a expression and sarcomere shortening, and reduced insulin-mediated phosphorylation of Akt in primary rat cardiomyocytes [123]. Outstandingly, in T2DM patients, plasma activin A levels were inversely associated with myocardial glucose metabolism, and positively with left ventricular mass/volume-ratio, reflecting a potential detrimental role of this molecule in early human diabetic cardiomyopathy [126].

#### Myocardial pro-steatosis biomarkers for DCM

Fat accumulation in the myocardium may be a protective response to provide a store of fuel for subsequent oxidation and to prevent exposure to toxic lipid metabolites such as ceramides. However, in DCM, a chronic imbalance of lipid storage versus lipid oxidation may lead to mechanical dysfunction [127]. Quantification of lipid droplets in cardiomyocytes by MRI has shown a direct measurement of myocardial steatosis, but it was not independently associated with diastolic dysfunction, possibly because of motion artefacts owing to contraction and breathing [128]. Thus, pro-steatosis factors released from heart may be useful for early DCM identification.

Hfabp is a cardiac cytosolic protein that acts transporting fatty acids to the mitochondria, for degradation and ATP consecution (Fig. 1). After lipid delivery, Hfabp is up-regulated and located at the sarcolemma but remains undetectable in serum of healthy subjects [129]. However, after myocardial injury such as myocardial infarction, systolic dysfunction or HF, Hfabp can be released to the plasma [129–131]. Interestingly, the presence of Hfabp has been observed in early cardiac injury of T2DM patients [132]. In addition, Shearer et al. demonstrated a correlation between Hfabp plasma levels and the severity of cardiac insulin resistance of T2DM mice [133].

#### Myocardial pro-fibrotic biomarkers for DCM

Cardiac transforming growth factor beta (TGFβ) has been associated with the fibrotic degree in DCM [134]. TGFβ activates Smad signaling to increase expression of extracellular matrix (ECM) proteins and reducing ECM-degradative enzymes [131]. Myocardial ECM is a complex network of fibrillar collagen types I and III (as well as less abundant collagen types IV, V and VI), and fibronectin, laminin, elastin, fibrillin, and proteoglycans. ECM mediates the mechanical connection among cardiomyocytes, fibroblasts and blood vessels within the myocardium, transmits extracellular mechanical signals, supports cell migration and endows architecture and integrity (Fig. 1). Importantly, diastolic dysfunction was ameliorated by inhibition of TGFβ in experimental T2DM, suggesting a central role of TGFβ-signalling in DCM pathogeny [135]. In this sense, a TGFβ mediator, Smad-3, has been also implicated cardiac hypertrophy, fibrosis, and diastolic dysfunction in T2DM mice [136]. Interestingly, an up-regulation of plasma TGFβ has been observed in diabetic patients with and without diastolic dysfunction. The highest levels were observed when both conditions coexisted [137]. Also, in T2DM mice, TGFβ was increased, independently of glucose levels, but associated with left ventricle collagen accumulation, myocardial stiffness and diastolic dysfunction [138].

In addition, cardiac fibroblasts could play a critical role in maintaining normal cardiac function, as well as in cardiac remodeling during pathological conditions such as DCM. Fibroblasts undergo phenotypic transition to myofibroblastic cells for ECM protein expression [139]. In this sense, collagens are synthesized as procollagen molecules, which are cleaved after secretion to become actives. This process is enhanced by the TGFβ/Smad pathway among other growth factors, such as PDGF and FGF. Cardiac fibroblasts also produce degradative proteins for ECM (i.e., metalloproteinases; MMPs) and their corresponding inhibitors (tissue inhibitors of metalloproteinases; TIMPs) to maintain an adequate balance between ECM deposition and degradation (Fig. 1). MMP-1, -2, -3 and -9 degrade collagens type-I, -II, -III and -IV. MMP-3 and -7 lyse laminin, proteoglycans, basal collagen membrane, and fibronectin [140]. However, MMPs can be inhibited by TIMPs and the MMPs/TIMPs ratio may indicate the grade of ECM degradation [141]. Thus, collagen synthesis and turnover could be useful for DCM recognition. In this sense, a relationship between increased procollagen type-1 propeptide (PINP) levels and the presence of diastolic dysfunction was unveiled in early T2DM patients [142]. In these patients, an elevation of serum MMP-7 was also demonstrated [141]. Similarly, in experimental T1DM, low expression of plasma MMP-2 has been observed in parallel with cardiac fibrosis and diastolic dysfunction [143]. In T2DM rats, decreased MMP-2 plasma activity, and elevated TIMP-2 levels were described in association with cardiac fibrosis [144].

A more forthcoming biomarker may be linked to the fibrotic, insulin-resistance and hypertrophic components of DCM (Fig. 1). Insulin-like growth factor binding protein-7 (IGFBP-7) is a modulator of insulin receptor activity by interaction with insulin growth factor-1 (IGF-1). IGFBP-7 has been evidenced as a serum biomarker for diastolic dysfunction associated with vascular remodelling and cardiac hypertrophy and fibrosis, in the metabolic syndrome [145]. A positive correlation between IGFBP7 levels and diastolic dysfunction was also detected in diabetic patients with reduced EF (detected by 2D-Echo) and increased collagen deposition [146].

## Conclusions and perspectives

Ideally, the presence of DCM should be investigated in both T1DM and T2DM patients when still asymptomatic. Thus, alterations in cardiac metabolism and myocardial strain in the myocardium, could be identified as early events in DCM. For this purpose, Phase-MRI could quantify metabolic disruptions, in spite of potential claustrophobia or incompatibility with pacemakers in some patients, and 2D/3D-STE may detect longitudinal and circumferential myocardial strains. In addition, imaging approaches may be complemented with a minimally-invasive quantification of specific plasma biomarkers. Previous reports showed that a quantification of NT-proBNP combined with 2D-Echo-Döppler imaging was more reliable to diagnose DCM in T2DM patients than any of them alone [147]. Furthermore, plasma molecules related to primary myocardial changes in DCM could be addressed. In particular, those related with hypertrophy, contractile alteration and steatosis such as CT-1, activin A and Hfabp, respectively. Additionally, IGFBP-7 plasma levels may inform about the insulin-resistance, hypertrophy and fibrotic status of the heart. However, key values or ranges of all these parameters have not been established yet for DCM patients, according with their sex, type of diabetes, and clinical history.

Moreover, early diagnosis of DCM could allow not only a reliable assessment of this pathology, but also, the use of preventive strategies to slow or avoid overt heart failure. For example, a DPP-4 inhibitor triggered beneficial effects on fatty acid uptake and oxidation, and prevention of diastolic dysfunction of T1DM rats [148]. In this regard, further investigations are needed for the discovery of new activated molecular pathways and mediators. Interestingly, noncoding RNA molecules such as microRNAs could play a role in cell-to-cell communications in different stages of DCM through their release into circulation [149]. In particular, a decrease of miR-133a may mediate diabetes-induced hypertrophy in mice, which resulted in upregulation of pro-hypertrophic transcription factors SGK1 and IGFR1 [150]. Also, miR-133a has been involved in GLUT4 regulation and glucose uptake in cardiomyocytes [151], and promotion of cardiac fibrosis via CTGF increase [152]. Further research in human DCM might also suggest miR-133a as a soluble biomarker for DCM. In this regard, another non-coding circulating RNA, long intergenic non-coding RNA predicting cardiac remodelling (lipcar), was positively associated with diastolic dysfunction in T2DM patients even before than NT-proBNP and c-reactive protein [153]. Thus, together with Phase-MRI or 2D/3D-STE imaging approaches, epigenetic molecules may complete a panel of predictive biomarkers for cardiac dysfunction in diabetic patients.
